# Diabetic Cardiomyopathy: Current and Future Therapies. Beyond Glycemic Control

**DOI:** 10.3389/fphys.2018.01514

**Published:** 2018-10-30

**Authors:** Giulia Borghetti, Dirk von Lewinski, Deborah M. Eaton, Harald Sourij, Steven R. Houser, Markus Wallner

**Affiliations:** ^1^Cardiovascular Research Center, Lewis Katz School of Medicine, Temple University, Philadelphia, PA, United States; ^2^Division of Cardiology, Department of Internal Medicine, Medical University of Graz, Graz, Austria; ^3^Division of Endocrinology and Diabetology, Department of Internal Medicine, Medical University of Graz, Graz, Austria

**Keywords:** diabetic cardiomyopathy, anti-hyperglycemic drug, SGLT-2 inhibitors, incretin-based therapy, heart failure, pathogenesis, treatment

## Abstract

Diabetes mellitus and the associated complications represent a global burden on human health and economics. Cardiovascular diseases are the leading cause of death in diabetic patients, who have a 2–5 times higher risk of developing heart failure than age-matched non-diabetic patients, independent of other comorbidities. Diabetic cardiomyopathy is defined as the presence of abnormal cardiac structure and performance in the absence of other cardiac risk factors, such coronary artery disease, hypertension, and significant valvular disease. Hyperglycemia, hyperinsulinemia, and insulin resistance mediate the pathological remodeling of the heart, characterized by left ventricle concentric hypertrophy and perivascular and interstitial fibrosis leading to diastolic dysfunction. A change in the metabolic status, impaired calcium homeostasis and energy production, increased inflammation and oxidative stress, as well as an accumulation of advanced glycation end products are among the mechanisms implicated in the pathogenesis of diabetic cardiomyopathy. Despite a growing interest in the pathophysiology of diabetic cardiomyopathy, there are no specific guidelines for diagnosing patients or structuring a treatment strategy in clinical practice. Anti-hyperglycemic drugs are crucial in the management of diabetes by effectively reducing microvascular complications, preventing renal failure, retinopathy, and nerve damage. Interestingly, several drugs currently in use can improve cardiac health beyond their ability to control glycemia. GLP-1 receptor agonists and sodium-glucose co-transporter 2 inhibitors have been shown to have a beneficial effect on the cardiovascular system through a direct effect on myocardium, beyond their ability to lower blood glucose levels. In recent years, great improvements have been made toward the possibility of modulating the expression of specific cardiac genes or non-coding RNAs *in vivo* for therapeutic purpose, opening up the possibility to regulate the expression of key players in the development/progression of diabetic cardiomyopathy. This review summarizes the pathogenesis of diabetic cardiomyopathy, with particular focus on structural and molecular abnormalities occurring during its progression, as well as both current and potential future therapies.

## Introduction

Diabetes mellitus is a major public health problem and represents a huge health concern for the global population. In 2010, 285 million people were affected, and this number is estimated to increase to almost 700 million people by 2040 (Shaw et al., 2010). Type 2 diabetes (T2DM) is a chronic metabolic disorder characterized by hyperglycemia and insulin resistance, also representing one of the major risks for developing heart failure (HF) (Schocken et al., 2008). In 1974, the Framingham study showed that diabetic patients have a 2–5 times higher risk of developing HF than age-matched, non-diabetic patients, and independent of other comorbidities. This suggests a specific intrinsic mechanism that drives the pathological cardiac remodeling in this population (Kannel et al., 1974). The United Kingdom Prospective Diabetes Study (Group) indicated an association between the risk of cardiovascular complications and glycemia, observing that for every 1% decrease in HbA1c there was an 18% reduction in myocardial infarction (MI) events (Group, U. P. D. S. U., 1998).

Heart failure is a multifactorial disease in diabetic patients. Both type 1 diabetes mellitus (T1DM) and T2DM are associated with an increase in macrovascular and microvascular dysfunction, resulting in ischemic events and altered vascular permeability (Krentz et al., 2007; Calcutt et al., 2009). Atherosclerosis and hypertension are often present in diabetic patients and contribute to coronary artery disease (CAD) and peripheral vascular disease, both of which affect the heart. However, besides these well-known pathological triggers, diabetes contributes to the development of HF through a more disease-specific variety of mechanisms, which are mostly driven by hyperglycemia, hyperinsulinemia, metabolic changes, and oxidative stress (Davidoff et al., 2004).

The aim of this review is to summarize molecular, structural, and functional changes occurring during the pathogenesis of diabetic cardiomyopathy. We will discuss management strategies, with particular focus on the therapeutic effect of glucose lowering drugs on HF development/progression, merging basic research and clinical observations. Emerging potential new targets and future prospects to improve the cardiovascular health of diabetic patients will be discussed as well.

## The Pathogenesis of Diabetic Cardiomyopathy

**FIGURE 1:**
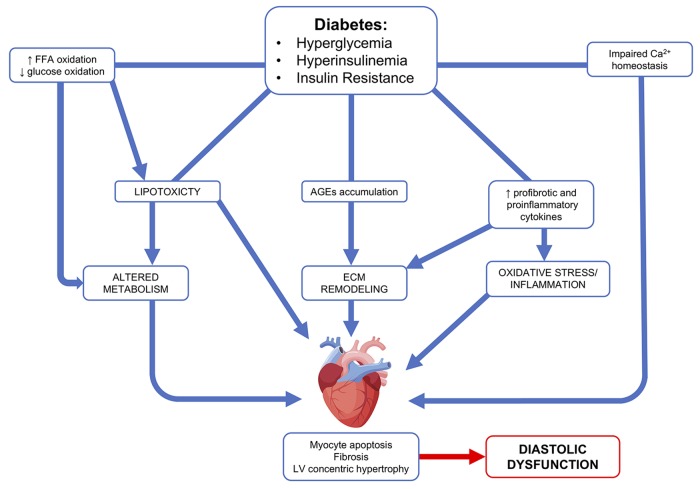
Diabetic cardiomyopathy pathophysiological mechanisms. Hyperglycemia, hyperinsulinemia, and insulin resistance lead to an increase in free fatty acid (FFA) oxidation, profibrotic and proinflammatory cytokines, as well as an accumulation of advanced glycation end products (AGEs). These abnormalities lead to altered metabolism, extracellular remodeling, oxidative stress, and inflammation. Ultimately, this leads to cardiac effects, such as myocyte apoptosis, fibrosis, and LV concentric hypertrophy.

Diabetic cardiomyopathy is defined as the existence of abnormal cardiac structure and performance in the absence of other cardiac risk factors, such CAD, hypertension, and significant valvular disease (Jia et al., 2018). It was first described more than four decades ago (Rubler et al., 1972), with hyperglycemia and impaired cardiac insulin signaling pathway having pivotal roles in its progression/onset (Jia et al., 2018). Clinically, the diabetic heart is characterized by diastolic dysfunction with preserved ejection fraction. These alterations are caused by the pathological remodeling of the heart. Increases in interstitial and perivascular fibrosis, as well as left ventricle (LV) hypertrophy are structural hallmarks associated with the diabetic heart (Tate et al., 2017). However, the underlying pathogenic mechanisms remain unclear; it includes but is not limited to abnormal extracellular matrix (Perge et al., 2017) deposition, an increase in oxidative stress and inflammation, in conjunction with mitochondrial dysfunction, and changes in the metabolic profile and energy production (Isfort et al., 2014; De Rosa et al., 2018; Figure 1).

An increase in fibrosis is the result of an increase in collagen deposition coupled with abnormalities in ECM protein structure and turnover (Tate et al., 2017). In the diabetic heart, upregulation in the expression of profibrotic factors, such as transforming growth factor beta 1 and connective tissue growth factor, can cause abnormal ECM protein deposition (Mizushige et al., 2000; Way et al., 2002; D’Souza et al., 2011). At the same time, a decrease in the activity of the ECM-degrading enzyme metalloproteinase (Westermann et al., 2007) can lead to ECM accumulation. Hyperglycemia induces advanced glycation end product (AGEs) formation, as a result of a non-enzymatic binding between amine residues of proteins or lipids and sugars (Kilhovd et al., 1999; Goh and Cooper, 2008; Yamagishi et al., 2012). AGEs damaging potential is correlated with their ability to cross-link collagen molecules, which increases their resistance to proteolysis and slows down their turnover (Aronson, 2003). AGEs may also bind to receptor for advanced glycation end products on the cardiac cell membranes, further promoting both pro-fibrotic and pro-inflammatory signaling, and increasing the expression of oxidative stress mediators (Candido et al., 2003; Haidara et al., 2006; Yamagishi et al., 2012).

Left ventricle hypertrophy is the main morphological change observed in the diabetic heart. Echocardiograms of diabetic patient’s hearts have shown an increase in LV posterior and septal wall thickness (Eguchi et al., 2008). LV hypertrophy can occur as an adaptive response to elevated hemodynamic stress (Ritchie et al., 2009). However, this morphological change can also occur independent of pressure-overload in diabetic patient (Galderisi et al., 1991; Eguchi et al., 2008). LV hypertrophy develops as a result of myocyte hypertrophy, an increase in interstitial and perivascular fibrosis, and thickening of the myocardial capillary basement membrane (Voulgari et al., 2010; Velic et al., 2013).

Metabolic dysfunction, hyperinsulinemia, oxidative stress, and inflammation are among the prevalent causes of this increase in LV mass seen in diabetic patients (Huynh et al., 2014). High glucose levels have been found to induce an increase in cardiomyocyte size *in vitro* (Feng et al., 2008). The diabetic heart is characterized by an upregulation in hypertrophic gene expression, such as atrial natriuretic peptide (ANP), B-type natriuretic peptide (BNP), and B-myosin heavy chain (Candido et al., 2003; Chang et al., 2006; Connelly et al., 2007; Ritchie et al., 2007; Huynh et al., 2010). Hyperglycemia activates the systemic and intracardiac renin–angiotensin–aldosterone system pathway resulting in an increase of angiotensin II (Ang II) levels (Frustaci et al., 2000). Ang II stimulates proliferation of cardiac fibroblasts and cardiomyocyte hypertrophy (Kumar et al., 2012). High levels of plasma aldosterone and overexpression of the mineralocorticoid receptor, along with increased Ang II activity, can exacerbate insulin resistance, hyperlipidemia, and hypertension (Baudrand et al., 2016).

Under normal physiological conditions, the adult heart can use a variety of substrates to produce ATP, a phenomenon called “metabolic substrate flexibility”. Free fatty acids (FFAs) are the preferred energy substrate of the adult heart, although other substrates, such as glucose, lactate, ketone bodies, and select amino acids can be used (Jia et al., 2016). Hyperglycemia and insulin resistance lead to a complete loss of this flexibility. A decrease in glucose transporter type 4 recruitment to the sarcolemma reduces the ability to use glucose as an energy source. At the same time, an increase in FFAs released from adipose tissue and FFAs transporter translocation to the sarcolemma leads to an internalization of this substrate in the cardiomyocytes (Harmancey et al., 2012). The loss of metabolic flexibility and the increase in fatty acid oxidation results in a loss of efficiency between substrate use and ATP production in the diabetic heart (Levelt et al., 2018). The energy source switch is accompanied by impaired oxidative phosphorylation and boosted mitochondrial ROS generation. This increase in mitochondrial uncoupling leads to increased mitochondrial O_2_ consumption, but this is not accompanied by a proportional increase in ATP synthesis, leading to a decrease in cardiac energy efficiency (Bugger and Abel, 2010; Rider et al., 2013). Moreover, the inability to switch to glucose oxidation makes the heart susceptible to damage and dysfunction under hypoxic conditions, such as in myocardial ischemia (Stanley et al., 1997).

An excessive accumulation of FFAs is detrimental to cardiomyocytes, as they are not equipped to store lipids. This highlights the concept of lipotoxicity as a mechanism for the development of diabetic cardiomyopathy through a decrease in myocyte physiological autophagy and an increase in apoptosis (Mandavia et al., 2013; Levelt et al., 2018).

Besides lipotoxicity, oxidative stress and inflammation are also mechanisms that trigger programmed cell death. An increase in the number of apoptotic cardiomyocytes was found in biopsies from diabetic patients in comparison to non-diabetic patients (Kuethe et al., 2007). In myocardial tissue of diabetic patients, metabolic and oxidative stress cause an increase in sensitivity to Ca^2+^ of mitochondrial permeability transition pore, that result in cardiomyocytes autophagy and cardiac necrosis (Anderson et al., 2011).

Maladaptive proinflammatory response furthers the progression of diabetic cardiomyopathy. Diabetes causes immune cell migration in the myocardium and an increase in macrophage pro-inflammatory M1 polarization, whereas the M2 anti-inflammatory phenotype is decreased (Jia et al., 2015). Upregulation of several proinflammatory cytokines, such as tumor necrosis factor (TNFα), interleukins 6 and 8, and monocyte chemotactic protein 1 is characteristic of the diabetic heart. These cytokines affect several cardiac cell populations, including cardiomyocytes, endothelial cells, fibroblasts, and smooth muscle cells, which all contribute to pathological remodeling and oxidative stress (Jia et al., 2018).

Proteotoxic stress, caused by accumulation of misfolded proteins and/or proteasome inhibition, has recently been discovered as an additional pathologic trigger for the diabetic heart. The cardiac ubiquitin proteasome system (Kruk-Bachonko et al., 2017) is responsible for the maintenance of protein homeostasis by degrading the misfolded or oxidized proteins (Gilca et al., 2017). UPS dysfunction occurs early on in the onset of diabetic cardiomyopathy and promotes cardiac maladaptive remodeling, whereas increasing UPS activity through PA28α overexpression has been shown to reduce cardiac dysfunction in a STZ-induced diabetes model (Li et al., 2017).

In addition, calcium handling machinery is directly compromised, as has been found in several animal models of both T1DM and T2DM. Hyperglycemia correlates with enhanced spontaneous calcium release from the sarcoplasmic reticulum, reduced cytoplasmic Ca^2+^ clearance, decreased SR Ca^2+^ load, and prolongation of action potential duration (Belke et al., 2004; Sorrentino et al., 2017). Some of the molecular changes responsible for the dysfunctional calcium handling have been studied in animal models and include: lower activity levels of the sarco/endoplasmatic reticulum Ca^2+^ ATPase 2 (SERCA2a) (Belke et al., 2004) and sodium (Na^+^)–Ca^2+^ exchanger (NCX) (Chattou et al., 1999), impaired ryanodine receptor (RyR2) function (Yaras et al., 2005), and reduced phospholamban phosphorylation (Gando et al., 1997). All of these abnormalities contribute to the defective excitation–contraction coupling associates with diabetes (Lebeche et al., 2008).

## Diabetic Cardiomyopathy: Clinical Manifestation and Diagnosis

Cardiac remodeling occurs in several different phases during the progression of diabetic cardiomyopathy, which is often asymptomatic during the early stages. The pathogenesis starts at a subcellular level, as described above, and the clinical manifestation of this dysfunctional remodeling is hypertrophy. Concentric LV hypertrophy is a strong predictor of adverse cardiovascular outcomes (Bluemke et al., 2008). The correlation between diabetes and hypertrophy was once reported as a result of other secondary comorbidities, such as aging, obesity, and hypertension (Kuperstein et al., 2001). However, several studies have shown a direct correlation between T2DM and an increase in LV mass, independent of hypertension and body mass (Eguchi et al., 2008). The next pathophysiological change is the development of interstitial and perivascular fibrosis, which have been identified as a more advanced stage in the disease progression. Hypertrophy and fibrosis cause impaired relaxation and passive filling of the LV and LV diastolic stiffness. As previously mentioned, diastolic dysfunction represents a major functional abnormality in diabetic patients, which can be asymptomatic during the earlier stages. Systolic dysfunction is less frequent and develops only in a small percentage of patients in the later stages of diabetic cardiomyopathy (Palomer et al., 2018). Furthermore, diabetes-associated fibrosis, found in both T1DM and T2DM, may contribute to the development of atrial fibrillation and arrhythmic events (Russo and Frangogiannis, 2016).

Little is known about the difference in the pathogenesis of diabetic cardiomyopathy in T1DM vs. T2DM. Both types of diabetes affect cardiovascular health. A common denominator seems to be the development of diastolic dysfunction. However, clinical presentation of HF is relatively rare in T1DM in comparison to T2DM, which may be due to patients being younger and being treated with insulin (Miki et al., 2013). There are fewer studies correlating T1DM with hypertrophy and an increase in LV mass compared to T2DM. No studies have found myocardial steatosis in T1DM (Levelt et al., 2018), opposed to what has been found in the hearts of T2DM patients, where steatosis precedes the diastolic dysfunction (McGavock et al., 2007). It should be noted that the underlying mechanism of diabetic cardiomyopathy may be different between the two distinct types of diabetes. Both T1DM and T2DM are characterized by hyperglycemia and dyslipidemia, but only T2DM also have hyperinsulinemia. This could explain the difference in cardiac morphology and clinical features found in patients affected by T1DM vs. T2DM, as well as differences between animal models (Holscher et al., 2016).

Currently, there are no specific morphological changes, biochemical markers, or clinical manifestations needed to secure a diabetic cardiomyopathy diagnosis. This pathology is often asymptomatic throughout the early stages and usually overlaps with other complications in diabetic patients, making a definitive diagnosis challenging.

In the past 20 years, incredible improvements have been made in non-invasive imaging technologies, such as echocardiography and magnetic resonance imaging (MRI), which provide detailed information about cardiac morphology and functions (Lee and Kim, 2017). Both transmitral Doppler and Tissue Doppler imaging are used to quantify the functional myocardial abnormalities. The ratio between early passive transmitral inflow velocity (E) and velocity of the medial mitral annulus (e’) is a substitute for invasively measured left ventricular filling pressure and a reliable prognostic marker for diabetic patients (Levelt et al., 2018). Abnormalities in E/e’ correlate with the development of HF and increased mortality, independent of other risk factors, such as hypertension and CAD (From et al., 2010).

Magnetic resonance imaging is capable of detecting abnormalities in cardiac morphology more accurately than echocardiography. This technique allows us to acquire precious information on myocardial fibrosis, steatosis, LV mass, and diastolic function. Positron emission tomography has been used to assess myocardial metabolic abnormalities. These new imaging techniques could be incredibly helpful for diagnosing diabetic cardiomyopathy at very early stages, but they are still only used for research purposes due to their cost, time demand, and level of expertise required to interpret results (Palomer et al., 2018).

## Current Therapies: Novel Glucose-Lowering Drugs

Hyperglycemia and chronic sustained hyperinsulinemia cause microvascular complications leading to renal failure, retinopathy, and nerve damage. Thus, lowering blood glucose levels is fundamental in the treatment regimen for diabetes. However, several observational studies fail to demonstrate a reduction in HF hospitalizations in diabetic patients treated with anti-hyperglycemic therapy. Moreover, in 2007 a meta-analysis showed a potentially increased risk of MI with the glucose lowering drug rosiglitazone, highlighting the necessity of thoroughly assessing the safety of this drug on the cardiovascular system (Nissen and Wolski, 2007). For this reason, the Federal Drug Administration (Hussain et al., 2018) and European Medicine Agency now require cardiovascular outcome trials for newly developed anti-hyperglycemic drugs in order to gain approval. This new regulation has resulted in a high number of cardiovascular outcome trials and increased the availability of important information on the effect of these drugs on cardiovascular health. Interestingly, several drugs currently in use can improve cardiac health beyond their ability to control glycemia (von Lewinski et al., 2017). However, there is not much data available regarding the mechanisms by which these drugs exert their pleiotropic effects other than these clinical/epidemiological studies.

### GLP-1 Receptor Agonists

Glucagon-like peptide 1 (GLP-1) is a gut-derived peptide hormone primarily secreted after food intake. This so-called incretin has the ability to decrease glycemia by increasing the release of insulin and repressing glucagon expression in a glucose-dependent manner (Mojsov et al., 1986).

Glucagon-like peptide 1 receptor is a G-protein coupled receptor that catalyzes the conversion of ATP in cAMP upon activation. Increased cytosolic cAMP in β-pancreatic cells leads to insulin secretion (Holst, 2007). Besides this, activation of the GLP-1 receptor in different tissues leads to a broad spectrum of effects, including deceleration of gastric emptying, suppression of appetite with consequent weight loss, reduction of circulating lipoprotein, and a decrease in blood pressure. However, endogenous secreted GLP-1 [GLP-1 (7–36)] has a very short half-life, which is approximately 2–3 min in the circulation. This active isoform is rapidly degraded primarily by dipeptidyl peptidase-4 (DPP-4) to GLP-1 (9–36), a receptor antagonist. Thus, several synthetic GLP-1 receptor agonists (GLP-1RA_s_) have been developed to provide prolonged *in vivo* action and subsequently have beneficial effects for T2DM patient (Meier, 2012). These drugs are able to increase insulin release only in the context of hyperglycemia. GLP-1RA_s_ have, therefore, a very low risk of inducing severe hypoglycemia, which is detrimental to the health of diabetic patients subjected to glucose-lowering treatment because it has been associated with an increase in cardiovascular events and mortality in this population (Zoungas et al., 2010).

Thus, GLP-1RA_s_ potential cardioprotective effects are derived from their ability to attenuate established cardiovascular risk factors, such as hyperglycemia, obesity, high blood pressure, and dysfunctional lipid profile. However, a more direct effect of this drug on the myocardium cannot be excluded and represents an area of growing interest. Several studies have shown the presence of GLP-1 receptor in atrial tissue of rodents and non-human primates (Wohlfart et al., 2013; Pyke et al., 2014; Richards et al., 2014). Wallner et al. (2015) reported GLP-1 receptor expression in both human atrial and ventricular tissue, although exenatide, a GLP-1RA_s_, exerts its inotropic effect only on atrial myocardium. Several studies have shown a GLP-1R-dependent activation of Epac-2, which is then able to translocate to the membrane and increase ANP secretion (Kim et al., 2013) and troponin I phosphorylation, with a consequent increase in myocyte contractility (Cazorla et al., 2009). Treatment with GLP-1RA_s_ or infusion of exogenous GLP-1 has been shown to decrease infarct size and improve cardiac function in several animal models of ischemic heart disease (Bose et al., 2005; Timmers et al., 2009; Liu et al., 2010; DeNicola et al., 2014). Interestingly, a small pilot study involving diabetic and non-diabetic patients found that 72 h of intravenous GLP-1 infusion in patients undergoing percutaneous revascularization after MI improved cardiac function (Nikolaidis et al., 2004). GLP-1RA_s_ have been found to be beneficial for the endothelium as well: treatment with exenatide resulted in reduction of glucose-induced ROS generation and apoptosis in endothelial cells of diabetic rats, and stimulates proliferation and NO synthase activity in human endothelia cells (Ding and Zhang, 2012). Thus, the modulation of GLP-1 signaling has exiting potential as a treatment option for diabetic patients, which goes far beyond its ability to reduce hyperglycemia.

The cardiovascular safety of GLP-1RA_s_ has been evaluated in several randomized clinical trials. All recent outcome trials have proven non-inferiority as requested by the authorities. However, cardiovascular outcome was heterogeneous between the trials with some neutral ones on the one side and others even proving superiority (Table 1).

**Table 1:** Principle characteristics of clinical trials evaluating effect of diabetes treatments on heart failure/cardiovascular outcomes (2010–2019).

Drug class	Name of drug	NCT identifier	Study name	Results	Clinical Trial Phase (total # of patients)	Trial duration
GLP-1 receptor agonists	Lixisenatide vs. placebo	NCT01147250	ELIXA	CV safety	Phase III (6,068)	25 months
	Exenatide vs. placebo	NCT01144338	EXSCEL	CV safety ↓All-cause mortality	Phase III (14,782)	38 months
	Liraglutide vs. placebo	NCT01179048	LEADER	↓3-MACE ↓All-cause mortality	Phase III (9,340)	45 months
	Semaglutide vs. placebo	NCT01720446	SUSTAIN 6	↓3-MACE	Phase III (3,297)	25 months
DPP-4 inhibitors	Sitagliptin vs. placebo	NCT00790205	TECOS	CV safety	Phase III (14,761)	36 months
	Alogliptin vs. placebo	NCT00968708	EXAMINE	CV safety	Phase III (5,380)	18 months
	Saxagliptin vs. placebo	NCT01107886	SAVOR-TIMI 53	CV safety ↑In HHF	Phase IV (16,492)	25 months
	Linagliptin vs. Glimepiride	NCT01243424	CAROLINA	Results expected 2019	Phase III (6,115)	Ongoing
	Linagliptin vs. placebo	NCT01897532	CARMELINA	Results expected 2018	Phase IV (8,300)	54 months
SGLT2-inhibitors	Canagliflozin vs. placebo	NCT01032629	CANVAS	↓3-MACE ↓HFF ↑Lower extremity amputations	Phase III (10,142)	43 months
	Empagliflozin vs. placebo	NCT01131676	EMPA-REG OUTCOME	↓3-MACE ↓All-cause mortality ↓HHF	Phase III (7,020)	37 months


*CV safety = cardiovascular safety, 3-mace = 3-point major adverse cardiovascular events, HHF = hospitalized heart failure. Blue: CV safety, Red: negative results, Green: positive results, Gray: trial still in progress/results expected in future.*

ELIXA trail was the first study to evaluate the CV safety of lixisenatide in 6,068 diabetic patients who had recently been hospitalized for acute coronary syndrome. There were no significant differences in cardiovascular events or hospitalizations after 25 months of follow-up between the lixisenatide and placebo group. Thus, this study demonstrated that the use of this drug is safe in patients with T2DM and recent acute coronary syndrome (Pfeffer et al., 2015). The cardiovascular safety of liraglutide was evaluated in the LEADER trial, in which 9,340 diabetic patients at high risk for cardiovascular events (having established cardiovascular disease, either CAD or chronic HF, and/or cerebrovascular disease, peripheral vascular disease, and chronic kidney disease) were enrolled. This study showed not only the safety but also the beneficial cardiovascular effect of this drug. Liraglutide was associated with a significant reduction in the primary composite endpoint, which includes CV mortality, non-fatal MI, non-fatal stroke as well as in all-cause mortality. Hospitalizations for HF were not different between the liraglutide and the placebo group (Marso et al., 2016b). Interestingly, subgroup analysis revealed that patients with more severe kidney disease, older or with established cardiovascular disease may have greater benefit from liraglutide treatment in comparison with other patient groups (Roder, 2018).

In the much smaller SUSTAIN-6 trial, patients with T2DM and established cardiovascular and renal disease were enrolled and randomized to receive either the longer acting semaglutide or placebo. The inclusion criteria were very similar to the LEADER study and the follow-up was 25 months. This trial, designed as a non-inferiority safety study, revealed that treatment with semaglutide decreased the combined primary outcome (cardiovascular death, non-fatal MI, and non-fatal stroke), however, there was no difference in the secondary endpoint of cardiovascular death alone (Marso et al., 2016a). The results of the primary outcome were driven by a reduction in non-fatal MI and stroke. However, in light of its smaller non-inferiority design, this trial may underestimate the difference in CV mortality compared with the much larger LEADER trial (Asleh et al., 2018).

The results of the large-scale EXSCEL trial were recently presented. Treatment with exenatide failed to demonstrate a significant reduction in the primary composite including death from cardiovascular causes, non-fatal MI, and non-fatal stroke (Holman et al., 2017). However, exenatide significantly reduced the secondary outcome of all-cause mortality. The reason for this divergence in results are still not fully understood, but the diabetic population studied in EXSCEL was more heterogeneous in terms of age and CV risks with the respect of the ones analyzed in the other trials. Moreover, a shorter follow-up period, lower baseline HbA1c level, a high discontinuation rate, together with a more frequent use of SGLT-2 inhibitor in the placebo group (Roder, 2018), could at least partly explain why there was no difference in CV events among treated and placebo group. Further studies are needed to determine which population of diabetic patients may benefit most from this therapy and investigate mechanisms leading to improved CV outcomes (Table 1).

### DPP-4 Inhibitors

Dipeptidyl peptidase-4 is expressed in most parts of cells/tissue and exhibits exopeptidase activity against GLP-1 and several other peptide hormones and chemokines. Thus its activity is not only limited to glucose metabolism but also regulates several processes, including inflammation, vascular function, cell homing, and survival (Mulvihill and Drucker, 2014).

Dipeptidyl peptidase-4 plasma activity correlates with cardiac dysfunction in humans and experimental models of HF (dos Santos et al., 2013), indicating a direct link between the DPP-system and cardiovascular health. In various models of HF, DPP-4 inhibition has improved ventricular remodeling, severity of HF, and even survival (Shigeta et al., 2012; Takahashi et al., 2013). Interestingly, a small study conducted with non-diabetic patients affected by non-ischemic myopathy has shown that the use of a DPP-inhibitor increases myocardial glucose uptake, opening up the possibility that this compound could be potentially beneficial in the progression of diabetic cardiomyopathy as well (Witteles et al., 2012). DPP-4 inhibitors prevent cardiac diastolic dysfunction by attenuating fibrosis and oxidative stress in mouse models of insulin resistance and obesity (Bostick et al., 2014).

Therefore, it was surprising that clinical outcomes with DPP-4 inhibitors showed heterogeneous and partially negative effects (or no effect) in cardiovascular health in large outcome trials, despite several smaller studies and translational data from animal models have suggested potential beneficial effects. Three DPP-4 inhibitors have been tested in large clinical trials: sitagliptin, alogliptin, and saxagliptin (Table 1). The use of these drugs was not associated with any increase (but neither a decrease) in the composite primary outcome, including CV mortality, non-fatal MI, and non-fatal stroke with hazard ratios very close to 1.00. However, HF hospitalization was modestly yet still significantly increased by 27% in the SAVOR-TIMI trial with saxagliptin (3.5% for saxagliptin vs. 2.8% for placebo). A similar, although not statistically significant trend could be detected in EXAMINE trial with 3.1% HF hospitalizations in the alogliptin group vs. 2.9% in the placebo group, whereas no difference was observed in the TECOS trial with an incidence of 3.1% for hospitalizations for HF in the sitagliptin and placebo group, respectively (Scirica et al., 2013; White et al., 2013; Green et al., 2015). The observation of increased HF hospitalization did not result in elevated mortality, but it was even more pronounced in subgroups with impaired renal function. More extensive data will soon be available from the outcome data for linagliptin from the CARMELINA and CAROLINA trials. No large outcome trial was performed using vildagliptin, which is not marketed for sale in the United States. These controversial data have been the starting point for contradicting interpretations of recent meta-analyses, ranging from no increase in the risk for the hospitalization of HF after DPP-4 inhibitor use (Savarese et al., 2016) to an increased risk (Kongwatcharapong et al., 2016), indicating that there are considerable differences between substances within the class of DPP-4 inhibitors. Moreover, DPP-4 inhibitors could have a different effect in different populations (relatively “healthy” diabetic population vs. higher risk subjects). However, sub-group analyses failed to identify a specific population in which beneficial cardiovascular effect were evident (Luconi et al., 2017).

Dipeptidyl peptidase-4 inhibitors have been shown to have an impact on various organ systems, including the heart, kidneys, vascular system, and the neuroendocrine system. It impacts hormones or second messengers like BNP, stromal cell-derived factor 1, neuropeptide Y (NPY), and substance P, causing partially activation of the sympathetic nervous system and stimulation of β-adrenergic receptors (Packer, 2018). However, long-term DPP-4 inhibitor treatment in a diabetic mouse model undergoing transverse aortic constriction resulted in an impairment of cardiac function due to an increase in proinflammatory and profibrotic gene expression (Mulvihill et al., 2016). Thus, upregulation of inflammatory cytokines could play a role in the potential interplay of DPP-4 inhibitors and increased hospitalization for HF (Luconi et al., 2017).

A recent study has shown that DPP-4 is absent in cardiomyocytes, but saxagliptin is internalized in these cells where it inhibits SERCA2a/Ca^2+^/Calmodulin-dependent protein kinase II/phospholamban axis and reduces Ca^2+^-extrusion *via* NCX. This results in depleted SR Ca^2+^-content and cytosolic Ca^2+^-overload, as seen in HF, which consequently leads to impaired myocardial function. Moreover, saxagliptin induced prolonged action potential duration and consequently QTc interval *via* reduced protein-kinase C-mediated delayed rectifier K^+^ current (Koyani et al., 2017).

Some of the drugs used in the clinic also inhibit DPP-8 and to a lesser extent DPP-9. These two DPPs are also located in the cytosol of cardiomyocytes and might therefore directly affect myocardial function, energetics, or metabolism. DPP-8 and DPP-9 were reported to have an impact on cellular homeostasis and energy metabolism *via* cytosolic calreticulin and adenylate kinase 2 (Wilson et al., 2013). In animal models, inhibition of DPP-8 or DPP-9 has even been described to cause a variety of symptoms ranging from alopecia over gastrointestinal disorders and blunted hematopoiesis to increased mortality in rats (Lankas et al., 2005). However, these side effects were not observed in the large clinical trials with saxagliptin (SAVOR-TIMI 53) or the previously completed smaller studies using vildagliptin, although these drugs also strongly inhibit DPP-8 and DPP9.

In conclusion, underlying signal transduction mechanisms derived from animal data must be discussed with caution. The possible therapeutic role of various DPP-4 inhibitors in patients with HF, and more specifically diabetic cardiomyopathy, is not yet fully understood.

### SGLT-2 Inhibitors

Since regulatory agencies began requiring cardiovascular outcome trials for newly developed anti-hyperglycemic drugs, the EMPA-REG-OUTCOME trial (Zinman et al., 2015) was the first to report remarkably improved CV outcomes including all-cause mortality in patients treated with one particular anti-diabetic drug (Table 1). This double-blind, placebo-controlled trial randomized 7,020 patients with T2DM at high CV risk to either once-daily empagliflozin treatment (10 or 25 mg) or placebo treatment. The investigators reported a significant 14% reduction of the combined primary endpoint encompassing CV death, non-fatal MI, and non-fatal stroke in patients treated with empagliflozin (pooled analysis) during a mean follow-up of 3.1 years, which was mainly driven by a reduction in CV deaths (hazard ratios, 0.62; 95% CI, 0.49–0.77; *P* < 0.001). Furthermore, a 35% relative reduction in the rate of HF hospitalization was observed in the empagliflozin group (*p* < 0.002). The CANVAS program (Neal et al., 2017a), which analyzed data from two sister trials, CANVAS and CANVAS-R (Neal et al., 2017b), included 10,142 patients with T2DM and a high CV risk and was designed to study the CV safety and efficacy of canagliflozin (SGLT2i). Consistent with the findings from the EMPA-REG-OUTCOME trial, canagliflozin significantly reduced the rate of primary outcome events (composite of CV death, non-fatal MI, and stroke) by 14% and HF hospitalization by 33%. However, all-cause and CV mortality were not significantly reduced by canagliflozin. The cardiovascular outcome trial investigating the effects of dapagliflozin has not been reported yet, however, the CVD-REAL (comparative effectiveness of cardiovascular outcomes in new users of SGLT-2 inhibitors) although not being a randomized controlled trial also suggests a reduction in mortality and HF hospitalization the real world setting, confirming the findings from the EMPA-REG-OUTCOME trial and the CANVAS program, suggesting a potential drug class effect at least on some of the outcome parameters (Kosiborod et al., 2017). However, the CANVAS program reported an unexpected increase in the risk of lower-extremity amputation in the canagliflozin-treated group. The mechanism of action is not understood, and furthermore it is not known if the higher amputation risk is specific to canagliflozin (Tanaka and Node, 2017). The striking and unexpected findings of SGLT2i regarding cardiovascular outcomes has initiated many discussions on how the cardiovascular benefits could be explained mechanistically, generating many hypotheses that are difficult to address with the limited data available. The described glucose lowering effect (Zinman et al., 2015), weight loss (Riggs et al., 2015), and blood pressure reduction (Baker et al., 2014), hemodynamic effects, direct vascular effects, osmotic diuresis, and natriuresis (Inzucchi et al., 2015; Butler et al., 2017) may contribute to the CV effects (Lytvyn et al., 2017). However, even a combination of these factors is unlikely to fully explain the results of EMPA-REG OUTCOME and the CANVAS program.

Recently, despite the lack of myocardial SGLT-2-expression, direct effects of the drug on heart muscle cells have even been suggested. There is evidence that the sodium hydrogen exchanger (NHE) may play an important role in the interplay of HF and diabetes since renal and cardiac isoforms of the NHE are upregulated in both conditions (Baartscheer et al., 2017; Packer, 2017). Inhibition of NHE by SGLT-2 inhibitors and modulation of intramyocardial Ca^2+^ and Na^+^ fluxes seems to have a beneficial impact on diastolic myocardial function (Baartscheer et al., 2017). Two recently published studies suggested that empagliflozin improves diastolic dysfunction in diabetic mouse models, which was linked to enhanced SERCA activity and anti-fibrotic effects (Habibi et al., 2017; Hammoudi et al., 2017).

Another potential explanation for the beneficial CV effects of SGLT-2 inhibition is a modulation of myocardial energy metabolism (Ferrannini et al., 2016). The myocardium of diabetic HF-patients loses the ability to properly oxidize fatty acids and metabolize glucose. It has been suggested that SGLT-2 inhibition slightly increases levels of ketone bodies independent of the presence of diabetes, which can then be oxidized in preference to fatty acids. This metabolic substrate shift might improve myocardial work efficiency and oxygen consumption (Bonner et al., 2015; Ferrannini et al., 2016; Al Jobori et al., 2017). However, the impact of SGLT-2 inhibitors on cardiac metabolism has not yet been carefully studied and it remains unclear how SGLT-2 inhibitors exert their beneficial CV effects through myocardial metabolism modulation (Ussher et al., 2016). Direct assessment of myocardial metabolism after SGLT-2 inhibition *in vivo* is challenging but would reveal new insights. Although there is a general consensus that SGLT-2 inhibition improves CV outcomes, several questions remain unanswered regarding the exact mechanisms of action, optimal timing, and whether or not these drugs would exert similar effects in non-diabetic patients with a high risk of CVD (Kaplan et al., 2018). Since the CV benefits of SGLT-2 inhibition seem to be independent of glucose control, SGLT-2 inhibitors might be both safe and effective in non-diabetic HF patients (Butler et al., 2017). Currently, several Phase III outcome trials with SGLT2 inhibitors in non-diabetic HF patients with preserved (HFpEF) and reduced ejection fraction (HFrEF) are planned and the field is eagerly awaiting the results.

## New Potential Targets for Future Therapies

### Gene Therapy

In recent years, great improvements have been made toward the possibility of modulating the expression of specific cardiac genes *in vivo* for therapeutic purpose. Thus, the idea to up or down-regulate the expression of key players in the development of diabetic cardiomyopathy may not be that far from an actual therapeutic approach.

The E3 ubiquitin ligase mitsugumin 3 expression is increased in the cardiac tissue of T2DM animal models. Cardiac-specific overexpression of this molecule leads to proteasomal degradation of both insulin receptor and IRS-1 resulting in insulin resistance (Liu et al., 2015). Furthermore, an increase in fibrosis was observed in transgenic mice, suggesting that the inhibition of E3 ubiquitin ligase mitsugumin 3 could represent an overall potential therapeutic strategy for the prevention of diabetic cardiomyopathy.

Forkhead box-containing protein 1, O subfamily (FoxO1) is another molecule involved in the regulation of IRS-1/Akt signaling pathway. Metabolic stress induces constant activation of FoxO1 that results in blunted Akt signaling and insulin resistance. Cardiomyocyte-specific deletion of FoxO1 rescued cardiac dysfunction and preserved insulin responsiveness in mice fed with a high-fat diet (Battiprolu et al., 2012). Thus, FoxO1 may be a potential target for future therapeutic approach.

Reduced chamber compliance is a hallmark change in HFpEF associated with T2DM and is partly due to altered phosphorylation of the structural sarcomeric protein titin with a consequent increase of cardiomyocytes stiffness. A recent study showed how treatment with Neuregulin1 (NRG-1) was able to rescue titin-based cardiomyocyte stiffening in diabetic mouse hearts (Hopf et al., 2018), *via* increased PKG and ERK1/2 activity and reduced PKCα activity, which reversed the changes in titin-phosphorylation associated with diabetes.

As mentioned above, diabetes is strongly associated with mitochondrial dysfunction. A recent study showed that diabetes disrupts mitochondrial proteomic signature. 99% of mitochondrial protein are encoded in the nucleus and then imported in this organelle by a complex, in which mitochondrial heat shock protein 70 (mtHsp70) is a key component. Diabetes correlates with a downregulation of this protein. Interestingly, mtHsp70 overexpression is able to restore cardiac function in diabetic mice through attenuation of mitochondrial dysfunction (Shepherd et al., 2018).

### Modulation of Oxidative Stress

Oxidative stress is one of the major contributors in the pathogenesis of the diabetic heart. A number of studies have evaluated different strategies to decrease ROS accumulation.

Sulforaphane, a dietary isothiocyanate compound is an activator of Nrf2, a transcription factor that regulates the expression of several antioxidant proteins. Sulforaphane treatment resulted in a decrease in ROS production in arterioles of diabetic mice (Velmurugan et al., 2013) and attenuated cardiac remodeling and dysfunction induced by high fat diet (Zhang et al., 2014).

Several studies conducted in the past 40 years have explored the efficacy of coenzyme Q_10_ in reducing oxidative stress and pathological remodeling of the heart (Huynh et al., 2014). Coenzyme Q_10_ treatment results in reduction of systolic and diastolic blood pressure in diabetic patients (Hodgson et al., 2002; Chew et al., 2008) acting as vasodilator. Moreover, supplementation with coenzyme Q_10_ decreases cardiac inflammation, fibrosis, and hypertrophy in mouse models of T1DM and T2DM (Huynh et al., 2010, 2013).

### miRNA and lncRNA-Based Treatment

miRNA-based treatment is a multi-target therapy causing simultaneous regulation of crucial pathways, making it an excellent candidate to modulate complex networks, such as those involved in the pathogenesis of diabetic cardiomyopathy.

miRNAs expression undergoes changes in the diabetic heart. miRNAs modulation can be a response to several pathological insults, including hyperglycemia, hyperinsulinemia, oxidative stress, and inflammation. Interestingly, glycemic control is unable to rescue hyperglycemia-induced alterations of miRNAs in the heart of streptozotocin-induced diabetic mice, suggesting that diabetic cardiomyopathy and the miRNA alterations associated with it, can progress despite normalization of blood glucose level (Costantino et al., 2016).

miR-1, the most expressed miRNA in the heart, constantly increases from the early to later phases of diabetic cardiomyopathy. It negatively regulates the expression of Pim1 and Bcl-2, which are anti-apoptotic and cardioprotective proteins. Remarkably, transfection with anti-miR1 activates pro-survival signals in cardiomyocytes and cardiac progenitor cell exposed to high glucose (Katare et al., 2011). On the other hand, miR-133a expression was drastically decreased in hearts of streptozotocin-induced diabetic mice. This downregulation correlates with the increase of fibrotic markers, such as TGFβ, fibronectin, and collagen. Interestingly, overexpression of miR-133a attenuates the development of fibrosis, suggesting that this miRNA could be a potential therapeutic target for diabetes-induced cardiac fibrosis and related cardiac dysfunction. Diabetes correlates with a decreased expression of miR-30c and miR-181a in human samples and animal models, and overexpression of these miRNAs in cardiomyocytes exposed to high glucose attenuated p53-induced apoptosis and hypertrophy (Raut et al., 2016).

Hyperglycemia reduces miR-146a expression in cardiac endothelial cells. Endothelial-specific overexpression of miR-146a attenuates the pathological remodeling in the diabetic heart and decreases the inflammatory response (Feng et al., 2017).

Finally, long non-coding RNA (lncRNAs) is a novel class of RNA that does not code for proteins and are important regulators of gene expression. Recent studies have found that diabetes correlates with an aberrant expression of these molecules (Raut and Khullar, 2018). Briefly, lncRNA-myocardial infarction-associated transcript (MIAT) expression is upregulated in models of diabetic cardiomyopathy and when knocked down, there are improvements in cardiac function and a decrease in cardiomyocyte apoptosis (Zhang et al., 2016; Zhou et al., 2017).

### Non-coding RNAs as Biomarkers

The possibility to easily detect different miRNAs in plasma and how stable they are opens up the opportunity to use these molecules as biomarkers for several pathologies, including diabetic cardiomyopathy (Marfella et al., 2013; Ono, 2015; Sardu et al., 2016a; de Lucia et al., 2017). The lack of specific biomarkers coupled with the fact that the earlier stages of this pathology are mostly asymptomatic makes detecting diabetic cardiomyopathy a challenge in clinical practice. miRNAs have been proposed as a new and very specific biomarker for several cardiovascular disease. A recent study determined that both circulating and cardiac miR-19b-3p and miR-181b-5p levels were associated with cardiac dysfunction during the development of diabetic cardiomyopathy in mice fed a high fat, suggesting that these miRNAs could be suitable biomarkers for this disease in asymptomatic diabetic patients (Copier et al., 2017). Furthermore, circulating miR-1 and miR-133a levels are associated with myocardial steatosis in T2DM patients, independent of confounding factors (de Gonzalo-Calvo et al., 2017).

A recent study conducted in patient suggested that also lncRNAs can be used as circulating biomarkers in diabetic cardiomyopathy. lncRNAs, such as long intergenic non-coding RNA predicting cardiac remodeling (LIPCAR), MIAT, and smooth muscle and endothelial cell-enriched migration/differentiation-associated (SENCR) were found to be independent predictors of diastolic dysfunction in diabetic patients (de Gonzalo-Calvo et al., 2016).

Further studies conducted in patients are needed to investigate the real potential of these molecules as biomarkers.

## Conclusion

Diabetes mellitus represents one of the greatest burdens on the global health care system and is one of the major risks for cardiovascular disease. Interestingly, cardiovascular risk is higher in diabetic women than in diabetic men, suggesting that diabetes affects cardiovascular health to greater degree in women. This difference is probably due to sex hormones and neurohormonal diversity, coupled with gender-specific activation of molecular pathway involved in the cardiac metabolism/remodeling (Toedebusch et al., 2018).

Diabetic cardiomyopathy is an important complication of diabetes and represents a distinct form of HF that is independent from other comorbidities. Its pathogenesis is not completely understood, although hyperglycemia and cardiac insulin resistance are key players. Further studies are vital for understanding the precise pathophysiological mechanism involved and to unravel the complex molecular and structural abnormalities leading to fibrosis, hypertrophy, mitochondrial dysfunction, steatosis, oxidative stress, impaired Ca^2+^ handling, inflammation, and metabolic switch observed in diabetic cardiomyopathy.

Despite a growing interest in the pathophysiology of the diabetic cardiomyopathy, there are no specific guidelines for diagnosing patients or structuring a treatment strategy in clinical practice. Currently, treatment plans are based on controlling the underlying diabetes and improving the risk factors associated with the progression of the cardiovascular disease. Several studies have found that glycemic control improved LV diastolic function in T2DM (Gaede et al., 2003; von Bibra et al., 2004). However, cardiovascular disease also occurs in diabetic patients who are well managed under treatment, highlighting the necessity for targeted therapeutic strategies for this population (Tate et al., 2017).

Changes in lifestyle favoring exercise, balanced caloric intake, anti-diabetic medications, lipid-lowering therapies, and the management of HF are the current treatment strategies available for these patients. Administration of beta-blockers, angiotensin converting enzyme inhibitors (ACEi)/angiotensin receptor blockers (ARB) are currently the standard treatment for chronic HF with or without diabetes. If patients remain symptomatic with LV EF <35%, it is recommended to add a mineralocorticoid receptor antagonist. If patients are still symptomatic, the ACEi/ARB should be replaced by an angiotensin receptor neprilysin inhibitor. Furthermore, cardiac resynchronization therapy should be considered in patients with sinus rhythm and wide QRS complex (≥130 ms) (Ponikowski et al., 2016; Sardu et al., 2016b, 2017). An in-depth description of therapeutic approaches for HF was beyond the scope of this review.

In this review, we focused mainly on anti-hyperglycemic drugs and their benefit on cardiovascular outcomes. Besides modulating diabetes as a cardiovascular risk, several new glucose lowering drugs have been found to have a direct effect on myocardial tissue. Although particularly promising, a more comprehensive analysis of cardiovascular outcome data together with translational studies is necessary to elucidate the benefit/risk and the mechanism of action of these glucose-optimizing agents. Specifically, understanding the real effect of glucose lowering drugs on diabetic cardiomyopathy based on cardiovascular outcome trial data is challenging. These trials were designed for safety purpose and include a very heterogeneous population of diabetic patients from the cardiovascular prospective. However, despite the lack of information on the direct effect on diabetic cardiomyopathy, these trials provide the suggestion of possible cardiovascular protection. Novel therapeutic approaches, including gene and non-coding RNA-based therapy, are currently being investigated in models and represent an exciting and promising new direction for treating diabetic patients. These therapies can eventually regulate common factors of diabetic cardiomyopathy and HF retaining a great potential in the cardiovascular field. However, further studies are needed to investigate the real translational potential in clinical practice.

## Author Contributions

All authors participated in writing and revising the content of the manuscript.

## Conflict of Interest Statement

HS received speaker’s honoraria from and is on the Advisory Board for Astra Zeneca, Boehringer Ingelheim, Eli Lilly, MSD, NovoNordisk, Sanofi-Aventis, and Takeda and received unrestricted Research grants from Astra Zeneca, Boehringer Ingelheim, MSD, and NovoNordisk. DvL received funding by Boehringer to conduct the EMMY-Trial. The remaining authors declare that the research was conducted in the absence of any commercial or financial relationships that could be construed as a potential conflict of interest.
